# Interpersonal Support, Emotional Intelligence and Family Function in Adolescence

**DOI:** 10.3390/ijerph18105145

**Published:** 2021-05-12

**Authors:** Ana Belén Barragán Martín, María del Mar Molero Jurado, María del Carmen Pérez-Fuentes, Nieves Fátima Oropesa Ruiz, África Martos Martínez, María del Mar Simón Márquez, José Jesús Gázquez Linares

**Affiliations:** 1 Department of Psychology, Faculty of Psychology, University of Almería, 04120 Almería, Spain; abm410@ual.es (A.B.B.M.); mmj130@ual.es (M.d.M.M.J.); mpf421@ual.es (M.d.C.P.-F.); foropesa@ual.es (N.F.O.R.); msm112@ual.es (M.d.M.S.M.); jlinares@ual.es (J.J.G.L.); 2 Department of Psychology, Universidad Autónoma de Chile, Providencia 7500000, Chile

**Keywords:** adolescence, social support, familiar functioning, emotional intelligence

## Abstract

Background: During adolescence, although the peer group exerts a strong influence on how the individual thinks and feels and on personal social values, the family still exerts a sustaining and supporting role. This study analyzed the relationships established between family function, emotional intelligence and perceived interpersonal support in adolescence. Method: The sample was made up of 1287 high school students aged 14 to 18 (*M* = 15.11; *SD* = 0.91) in the province of Almeria (Spain). Results: The results showed moderate correlations between the intrapersonal emotional intelligence dimension and perceived availability of support (advice or orientation), and between the mood dimension of emotional intelligence and the three interpersonal support dimensions (appraisal, belonging and tangible). In addition, significant positive correlations were found between family function and the intrapersonal and mood dimensions of emotional intelligence, with medium and large effect sizes, respectively. Apart from that, the data revealed that students who could count on a more functional family referred to high empathy and acceptance by others and greater support in material or financial matters, followed by those with moderate family function. In addition, students from homes with severely dysfunctional families perceived less available support. Finally, students who said they could count on strong family function also scored higher on the intrapersonal factor of emotional intelligence. Conclusions: The implications of these findings for the development of emotional intelligence in early adolescence are discussed from the family context, considering the relationship between emotional intelligence and social support.

## 1. Introduction

Adolescence has been established as a critical period of psychosocial development. During this period, young people must progress in the formation of a stable personality, the acquisition of their own identity and learning relational and coping mechanisms necessary for adulthood. In this sense, relations with parents and schoolmates influence the psychosocial maturity of youths [1]. The ways the members of a family group relate to each other characterize family function and dynamics. During adolescence, although the peer group exerts a strong influence on the individual’s thinking, feeling and social values, the family continues to exert a sustaining and supporting role. This study focused on the relationships between family function, emotional intelligence and perceived interpersonal support in adolescence. This study concentrates in particular on late adolescence (from age 14), when the most abrupt physical and cognitive changes have already taken place, but their body and mind continue developing. During these years, risks and adolescent idealism combine with the need to establish their identity and construct their own world, while they begin to actively participate on all social levels [2].

Family functions during adolescence, as in other evolutionary periods, could be summarized as: socialization and control of behavior, affective and emotional support, protection and economic support, and educational and recreational support [3,4,5,6]. While emotional intelligence is the ability to understand and manage our emotions and those of others appropriately and satisfactorily, we know that emotional intelligence is not innate, but can be educated. In this sense, and in regard to the role of the family in affective development, Hamarta, Deniz and Saltali [7] found that a secure attachment style predicted all the dimensions of emotional intelligence (intrapersonal, interpersonal, adaptability, stress management and general mood). Therefore, the family is an essential context for working on emotions from the first months of life, which invites the creation of spaces for communication. Through communication and the basically affective relationships established among family members, it also exerts a helping and supportive problem-solving function. During this stage of development, friends also have a role in emotional support in times of difficulty, are instrumental support in solving problems and are a source of information on subjects of interest. Such relationships are characterized by being more symmetric and based on reciprocity and mutual support. Before going on to describe the relationships between the psychological variables mentioned (interpersonal support, emotional intelligence and family function), however, it should be understood what is meant by each of these constructs, and some of the instruments most employed for their evaluation.

### 1.1. Interpersonal Support

The Social Support variable has been studied during adolescence [8,9]. It can be analyzed from a more structural perspective related to the size of the social network, to the number of social contacts and frequency with which these social relations are maintained, or from a more functional approach, referring to perceived empathy, acceptance or attention received from the social network itself [10]. One of the instruments most employed for evaluating perceived social support is the Interpersonal Support Evaluation List, consisting of 40 items, in its original version [11] and its brief 12-item version [12]. This shorter version of the instrument provides a global score on perceived social support as well as specific scores in three broad dimensions: appraisal, which includes perceived support insofar as orientation or advice; belonging, which refers mainly to perceived empathy, acceptance or concern by others; and tangible support, related to help received in material or economic matters. Support from friends, as mentioned above, is a very valuable resource for adolescents; however, to establish these links of friendship and to maintain them over time, they must possess certain socioemotional competencies [13,14,15]. Social support begins before birth, is consolidated with time based on interaction with parent figures, and later extended to friends, partner or coworkers [16]. At school, this variable has been related to grades, as demonstrated in the study by Hogan et al. [17], in which social support predicted the average grades of Australian adolescents in the sample.

### 1.2. Emotional Intelligence

Emotional Intelligence may be understood as an assortment of noncognitive skills, competencies and abilities that influence one’s capacity for dealing successfully with environmental demands and pressures [18]. In 1990, Salovey and Mayer [19] introduced the concept of emotional intelligence in the classroom, Gardner [20] compared emotional intelligence to the development of intrapersonal and interpersonal intelligence, and in 1995, Goleman [21] wrote his major work entitled *Emotional Intelligence,* which led to the concept’s diffusion. All these authors shared the idea that being emotionally intelligent involved the capacity for approaching, understanding and feeling one’s own emotions and those of others, and being able to act consequently. According to Bar-On’s trait model, the Emotional Intelligence Inventory (EQ-i) [18,22,23,24,25] can be used to measure an emotional quotient appropriately [26]. It classifies emotional intelligence in five main dimensions: intrapersonal (emotional understanding of oneself, assertiveness, self-concept, self-realization and independence), interpersonal (empathy, social responsibility and interpersonal relations), stress management (stress tolerance and impulse control), adaptability (confirming reality, flexibility and problem-solving) and general mood (happiness and optimism). Emotional intelligence in adolescence can predict general self-concept [27,28], and is also related to more satisfaction with life [29] and better academic performance [17,30]. With respect to the relationship between emotional intelligence and gender, Baumgartner [31] found that in early adolescence, boys were more withdrawn and less friendly than girls who had higher scores in social intelligence components. Silveri, Tzilos, Pimentel and Yurgelun-Todd [32] found that girls scored lower in emotional intelligence and higher in stress management than boys.

### 1.3. Family Function

The great physical and psychological transformations rapidly undergone by adolescents are usually accompanied by changes in family function, and therefore, in this period of their life, family system functionality and social support are key elements which facilitate positive adolescent development [33]. Family function refers to the capacity of the family’s members to take up the psychological processes involved in assuming functions within the group. There are several instruments for evaluating family function. In general, these tests measure such variables as the capacity for problem-solving, communication, roles, the capacity for affective response, the capacity for affective participation, behavior control, cohesion and adaptability, family satisfaction, etc. [34,35,36,37,38,39,40,41,42,43]. Smilkstein [44] suggested that family function referred to adaptation and resources accumulated by members of a family throughout their lives.

One of the most widely used instruments for evaluating perceived family function is the Family APGAR [45,46]. This is a simple test, easily applied, which provides information on family function and enables family dysfunction to be identified. The following areas are evaluated from this perspective: capacity for *adaptation*, through the use of intra and extra familial resources to help solve problems at times of stress; capacity for *partnership* in decision-making and family responsibilities; *growth*, or physical and emotional maturation and self-fulfillment through mutual support and guidance of its members; *affection*, that is, whether the relationship is based on love and caring among the members of the family group; and capacity for *resolve*, solving the needs of its members, devoting them time and providing material and personal resources. 

### 1.4. Relationship of Family Function, Emotional Intelligence and Interpersonal Support

It holds true that the adolescent experiences more emotional wellbeing in a functional family system in which its members can count on social support. The literature seems to emphasize such a close relationship between perceived family function and mood in adolescence [47,48,49,50]. Pérez-Fuentes, Molero, Barragán and Gázquez [48] found a relationship between the emotional intelligence mood dimension and the perception of family function in adolescents. In this sense, some studies emphasize that the perception of family function influences the presence of depressive symptoms in adolescents [49,50], so less family functionality may be associated with depression in adolescence. According to Freed, Rubenstein, Daryanani, Olino and Alloy [47], family function, emotional clarity and depressive symptoms are strongly related constructs at various times during adolescence, which has important implications for intervention, especially within the family unit. There are few studies exploring the relationship between family function and social support. Among those we were able to review is the excellent study by Pérez et al. [49], which analyzed the relationship between family structure and functionality with social support and psychological distress in a sample of 386 adolescents. The results showed that the adolescent’s perception of family function influenced social support, taking drugs and alcohol and the presence of depressive symptoms. Lack of family support has also been studied with regard to certain maladjusted conducts during adolescence [51,52,53,54]. Thus, for example, negligent parenting is associated with negative consequences during this stage, such as violent behavior and control when dating [55], high alcohol consumption [56], becoming the victim or aggressor in cyberbullying [57], and problems with social integration [58]. Thus, an inadequate parenting style and family atmosphere are key to adolescent adjustment, as well as developing deviant behavior during adolescence.

### 1.5. Relationship between Emotional Intelligence and Interpersonal Support

Previous studies have analyzed the relationship between some aspects of emotional intelligence and the configuration and characteristics of social support networks in adolescence. Bar-On’s trait model of Emotional Intelligence emphasizes the fundamental role of emotions in interpersonal relations, where emotion management and control are essential for a beneficial social life [59]. Concerning emotional awareness, Rowsell et al. [14] studied the ability to identify emotions in the composition of social networks, and found that emotional awareness influences such composition in girls, as the social networks of girls were more likely to be made up of peers of the same gender when they scored high in identification with emotions, while when the scores were low, the girls tended to choose boys for interaction. However, these authors found no significant differences between boys and girls insofar as the number of friends or the amount of time spent with them. With regard to mood, Ciarrochi and Heaven [13], in a study with Australian adolescents, concluded that pessimistic adolescents were incapable of positively influencing their social relations, and therefore, tended not to take action for developing and maintaining social support networks. Other findings have shown that few socioemotional competencies (aggression in peer relations and low self-revelation) in adolescents was associated with losing friends [15]. Ciarrochi, Heaven and Supavadeeprasit [60] showed that low ability to identify emotions predicted more fear, less positive affect and lower quality and quantity of social support. This study also found that a low ability to identify emotions also predicted more sadness. Other studies have shown that social support and emotional intelligence are significantly related in both genders, where emotional intelligence predicted the different types of support in both boys and girls [61].

Based on the findings above, this study was intended to: (1) analyze the relationships between emotional intelligence, interpersonal support and family function; (2) find out whether there are significant gender differences between emotional intelligence factors, interpersonal support dimensions and family function; and (3) determine whether there are significant differences between the categories of family function in emotional intelligence components and interpersonal support. The hypotheses posed were the following: (1) there are significant gender differences in emotional intelligence; (2) there are significant gender differences in interpersonal support; (3) there are significant differences between gender and family function; (4) there are significant differences between emotional intelligence and interpersonal support; (5) there is a significant positive relationship between emotional intelligence and family function; (6) there is a significant positive relationship between interpersonal support and family function; (7) there are significant differences in the three groups of family function categories with regard to the dimensions of interpersonal support; and (8) there are significant differences between the three family function categories and the emotional intelligence factors.

## 2. Materials and Methods

### 2.1. Participants

A cross-sectional study was conducted with random cluster sampling. The sample was made up of a total of 1287 high school students from Almeria province (Spain), aged 14 to 18 (*M* = 15.11; *SD* = 0.91). Of these, 52.9% (*n* = 681) were girls with a mean age of 15.10 (*SD* = 0.88) and 47.1% (*n* = 606) were boys with a mean age of 15.12 (*SD* = 0.94).

### 2.2. Instruments

*Ad hoc questionnaire.* A questionnaire designed by the authors collected the sociodemographic variables (age, sex and course), and some questions on the relationships of students with their parents/guardians (“Evaluate your current relationship with your parent/guardian”).

*Interpersonal Support Evaluation List shortened version* [12]. The brief version of the 12-item perceived social support questionnaire was used. This provides information on three support dimensions: Appraisal Support (e.g., “I feel there is nobody I can share my most private worries and fears with”), Belonging Support (e.g., “I don’t often get invited to do things with others”), and Tangible Support (e.g., “If I were sick, I could easily find someone who would help me with my daily chores”). The items are answered on a four-point Likert-type scale (where 1 = definitely false and 4 = definitely true). In this study, the confidence intervals were optimum, and the Cronbach’s alpha and the McDonald’s omega were both 0.81. Additionally, for each of the subscales, reliability was: α = 0.63 and ω = 0.66 for Appraisal Support; α = 0.70 and ω = 0.71 for Belonging Support; α = 0.57 and ω = 0.58 for Tangible Support. This instrument has been applied to populations with similar characteristics, showing acceptable psychometric properties. Alghamdi, Aslam and Khan [62] found a reliability of α = 0.70.

*Brief Emotional Intelligence Inventory for Senior Citizens* (EQ-i-M20) [63]. In this study, the adaptation by Pérez-Fuentes, Gázquez, Mercader and Molero [64], validated and scaled in an adult Spanish population, was used. This inventory consists of 20 items distributed in five factors: Intrapersonal (e.g., “It is easy for me to tell people how I feel), Interpersonal (e.g., “I know how other people feel”), Stress Management (e.g., “I have a bad temper”), Adaptability (e.g., “It’s easy for me to understand new things”, and Mood (e.g., “I feel sure of myself”). The answers are rated on a four-point Likert-type scale. The Cronbach’s alpha for each of the scales was: 0.57 for the intrapersonal factor; 0.80 for interpersonal; 0.68 for stress management; 0.81 in adaptability; and 0.83 for general mood. For this sample, internal consistency of the instrument was α = 0.79 and ω = 0.77 and for each of the subscales the reliability was: α = 0.81 and ω = 0.81 for the Intrapersonal subscale; α = 0.57 and ω = 0.62 for the Interpersonal subscale; α = 0.77 and ω = 0.77 for Stress Management; α = 0.71 and ω = 0.71 for the Adaptability subscale; and α = 0.87 and ω = 0.87 for Mood.

*Family Function Scale* (APGAR) [45]. This study used the Spanish adaptation of the original version [46]. It is comprised of five items with three answer choices (0 = hardly ever, 1 = some of the time and 2 = almost always), which evaluate adaptation, growth, partnership, affect and resolve. The scale also provides three functionality categories: severely dysfunctional (0 to 3), moderately dysfunctional (4 to 6) and functional (6 or more). The Cronbach’s alpha was 0.84. The objective of this study was to analyze the validity and reliability of the Family APGAR family function questionnaire. As to the psychometric properties of the test, the reliability and validity were optimum [45]. In this study, the instrument’s reliability was adequate, with a Cronbach’s alpha of 0.77 and McDonald’s omega of 0.78.

### 2.3. Procedure

First, the principals at the eleven high schools were contacted to inform them of the study’s objectives, methods, use of data, and obtain their consent. The students were told that their participation was voluntary and they were given the instructions necessary for filling out the questionnaire on paper. Then, they were informed of the confidentiality and anonymity in handling data. Informed consent was received from parents/guardians and also from the participants themselves in compliance with the ethical standards of research. The study was approved by the University of Almeria Bioethics Committee (Ref: UALBIO2018/015).

### 2.4. Data Analysis

The SPSS statistics program [65] version 25.0 for Windows was used for data processing and analysis.

Normality tests were performed for the dependent variables, and values were significant (*p* < 0.05). However, in some cases, the use of parametric tests is rather resistant to deviations from normality [66], and according to the central limit theorem, more so the larger the sample size is. As the sample size increases (*n* > 200), the use of parametric tests is considered acceptable, even for very biased distributions [67].

First, a descriptive analysis was made. The Student’s *t* test for independent samples was used to find out whether there were any significant differences between emotional intelligence, interpersonal support and family function by gender, and to estimate the effect size, the Cohen’s *d* [68]. Then, the relationship between the three constructs was explored employing the Pearson’s *r*.

Finally, after the youths were grouped by family function categories following the guidelines of Bellón et al. [45], a Multivariate Analysis of Variance (MANOVA) was performed to find out whether there were any differences between the categories of family functionality with respect to the components of emotional intelligence and social support. Effect sizes were interpreted employing the criteria of Cohen [68], where the effect is small when *η_p_*² = 0.01 (*d* = 0.20), medium when *η_p_*² = 0.059 (*d* = 0.50) and large if *η_p_*² = 0.138 (*d* = 0.80). Furthermore, to analyze the relationship with each of the dependent variables individually, a univariate analysis (ANOVA) was done.

The McDonald’s omega coefficient was calculated [69] to test the reliability of the instruments following the proposal and guidelines of Ventura-León and Caycho [70].

## 3. Results

### 3.1. Subsection

Table 1 shows the results of the means of the emotional intelligence factors by gender, where girls (*M* = 11.99; *SD* = 2.75) scored statistically significantly higher on the Interpersonal factor (*t*_(1285)_ = −6.80; *p* < 0.001; *d* = 0.38) than boys (*M* = 11.18; *SD* = 2.17). However, the boys had statistically significantly higher scores than girls on the Stress Management (*t*_(1285)_ = 3.30; *p* < 0.01; *d* = 0.18), Adaptability (*t*_(1285)_ = 2.81; *p* < 0.01; *d* = 0.16) and Mood (*t*_(1285)_ = 6.08; *p* < 0.001; *d* = 0.34) factors.

In Interpersonal Support (see Table 2), statistically significant differences were found by gender in Appraisal Support (*t*_(1285)_ = −4.33; *p* < 0.001; *d* = 0.24), Belonging Support (*t*_(1285)_ = −4.39; *p* < 0.001; *d* = 0.25) and Tangible Support (*t*_(1285)_ = −4.44; *p* < 0.001; *d* = 0.25), where girls had higher mean scores than boys in all three dimensions.

Results on family function by gender found that although there were no differences between groups, boys (*M* = 7.49; *SD* = 2.27) had higher mean scores than the girls (*M* = 7.27; *SD* = 2.49) on family function (*t*_(1285)_ = 1.67; *p* = 0.09), resulting in a tendential *p*, between 0.05 and 0.10.

The results derived from the correlation analysis, as shown in Table 3, suggest that the intrapersonal factor of emotional intelligence correlated positively with all the intrapersonal support dimensions (Appraisal Support: *r* = 0.272; *p =* 0.000; Belonging Support: *r* = 0.193; *p =* 0.000; Tangible Support: *r* = 0.137; *p =* 0.000) and Family Function (*r* = 0.292; *p =* 0.000). In addition, the Interpersonal factor showed positive correlations with the dimensions of Interpersonal Support (Appraisal Support: *r* = 0.158; *p =* 0.000; Belonging Support: *r* = 0.142; *p =* 0.000; Tangible Support: *r* = 0.183; *p =* 0.000) and Family Function (*r* = 0.136; *p =* 0.000). Stress Management also correlated positively with Appraisal Support (*r* = 0.092; *p =* 0.001), Belonging Support (*r* = 0.077; *p =* 0.006), Tangible Support (*r* = 0.096; *p =* 0.001) and Family Function (*r* = 0.148; *p =* 0.000). The Adaptability factor showed a positive correlation with Appraisal Support (*r* = 0.146; *p =* 0.000), Belonging Support (*r* = 0.174; *p =* 0.000), Tangible Support (*r* = 0.200; *p =* 0.000) and Family Function (*r* = 0.166; *p =* 0.000). The Mood factor also positively correlated with the three dimensions of Interpersonal support (Appraisal Support: *r* = 0.290; *p =* 0.000) and Family Function (*r* = 0.414; *p =* 0.000). Family Function was positively correlated with Appraisal Support (*r* = 0.274; *p =* 0.000), Belonging Support (*r* = 0.218; *p =* 0.000) and Tangible Support (*r* = 0.227; *p =* 0.000).

### 3.2. Emotional Intelligence, Interpersonal Support and Family Function

In another vein, the students were grouped by family function categories into severely dysfunctional, moderately functional and highly functional families. After that, the differences in the interpersonal support dimensions and emotional intelligence factors were analyzed by these groups.

Homogeneity of covariance was examined using Box’s M test, and the null hypothesis was rejected (*M_Box_* = 64.49; *F*_(12, 489040)_ = 5.33; *p* < 0.001). The multivariate contrast showed significant differences (Lambda de Wilks = 0.93; *F* = 15.5876; *p* < 0.001; *η_p_*² = 0.035). In this case, there was a small effect.

Analyzing the relationship separately for each of the dependent variables, statistically significant differences were found between the family function categories for Appraisal Support (*F* = 36.07; *p* < 0.001), Belonging Support (*F* = 27.51; *p* < 0.001) and Tangible Support (*F* = 29.19; *p* < 0.001). Thus, students who had a highly functional family also had more Belonging and Tangible Support, followed by those with moderately functional families, while those with the lowest Appraisal Support had severely dysfunctional families (see Table 4).

Nevertheless, the post hoc analysis showed that the between-group differences were statistically significant for each of the interpersonal support subscales.

Homogeneity of covariance of the emotional intelligence factors was examined using the Box’s M, and the null hypothesis was rejected (*M_Box_* = 58.01; *F*_(30, 375259)_ = 1.91; *p* < 0.001). The multivariate significance of principle effects and interactions was evaluated with Wilks’ Lambda (Wilks’ Lambda = 0.829; *F* = 25.18; *p* < 0.001; *η_p_*² = 0.090). In this case, there was a medium effect.

Analyzing the relationships separately for each of the dependent variables, statistically significant differences were found between the family function categories in the Intrapersonal (*F* = 37.56; *p* < 0.001), Interpersonal (*F* = 7.28; *p* < 0.01), Stress Management (*F* = 18.23; *p* < 0.001), Adaptability (*F* = 9.79; *p* < 0.001) and Mood (*F* = 104.73; *p* < 0.001) dimensions (see Table 4).

However, the post hoc analysis showed that in the intrapersonal and mood factors, all the between-group differences were statistically significant, and that the high-functionality group scored significantly higher than the rest on the interpersonal, stress management and adaptability factors.

## 4. Discussion

This study attempted to show the importance of analyzing the relationships between emotional intelligence, interpersonal support and family function in a sample of high school students, to understand the role of family context, and in particular, certain variables of family function (adaptability, growth, partnership, affection and resolve) in the positive development of adolescents.

The first idea inferred from the analysis which we have just made of the comparisons between the emotional intelligence dimensions, interpersonal support and student gender is that although significant differences were found in these comparisons, with higher means by girls in the interpersonal dimension of emotional intelligence, and in the rest of the dimensions of emotional intelligence (stress management, adaptability and mood) in favor of boys, the effect size was small in all the comparisons. In interpersonal support, the significant differences found in all types of support (appraisal, belonging and tangible) were in favor of the girls, also with a small effect size. In addition, the differences were not statistically significant in the comparisons between global scores on family function and gender. Therefore, our study demonstrated that the role of gender in relation to emotional intelligence and interpersonal support was not especially determining for the development of emotional intelligence of high school students, nor with regard to social support. With respect to family function, the data cause our third research hypothesis, in which it was expected for there to be significant differences between gender and family function, to be rejected. Other studies have found results both in favor of girls and of boys in emotional intelligence [31,32], demonstrating the complexity of the phenomenon itself, which along with the many definitions and interpretations, favors disparity of results [71]. In this sense, it seems that the data on emotional intelligence and gender found in this study, as mentioned by Garaigordobil [72], show that there are slight differences between boys and girls in emotional intelligence in favor of the latter. However, she also says those differences could be the fruit of interaction of other variables, such as the influence of culture and education on new generations, and these relationships should be interpreted with caution. Whereas studies analyzing the role in social support and family function are rather scarce, in social support, the results of the study by Rowsell et al. [14] suggested that emotional skills were especially important to the configuration of social networks of girls, particularly with respect to emotional awareness (ability to recognize one’s own emotions and those of others) and especially in early adolescence. Similarly, the study conducted from an ecological approach by Núñez-Fadda et al. [73] concluded that the family, and especially parents, are essential to emotional socialization and learning skills for their adolescent children’s positive development in different contexts. To a great extent, parenting and socialization are influenced by the gender of their children, since they cut across perceptions, cognitions and actions, affecting them all. This could partially explain the results found, as the differences in emotional intelligence and social support by gender could be reflecting the effect of different parenting practices, although this would be independent of family functioning.

With respect to the relationship between the dimensions of emotional intelligence and interpersonal support, the data showed that the most important correlations were found between the intrapersonal factor (understanding one’s own emotions, assertiveness, self-concept and self-realization and independence) and appraisal support (advice or orientation) and between the mood (happiness and optimism) factor and the three interpersonal support dimensions (appraisal support, belonging support and tangible support), where the effect size was moderate in all the correlations, thus meeting our fourth research hypothesis.

Family participates in the affective and social development of its members, contributing to socialization. The affective relations children have with their main attachment figures in childhood continue later in adolescence and adulthood. In the study by Hamarta et al. [7], a secure attachment style predicted all the dimensions of emotional intelligence. Insofar as our fifth research hypothesis, in which it was expected to find significant positive relationships between the dimensions of emotional intelligence and family function, our main results showed a significant positive relationship between the intrapersonal emotional intelligence factor and family function with a moderate effect size, and between the mood factor and family function, with a large effect size. In the same direction, our data also reflected that students with highly functional families scored higher on the intrapersonal factor of emotional intelligence. In this sense, previous research is conclusive, reinforcing the role of family function in adolescent affective development [43,44,45,46,47,48,49,50].

The results supported our sixth research hypothesis concerning the relationship between family function and interpersonal support, insofar as the high scores in appraisal support correlated with high scores in family function, with a medium effect size. In the same direction, but this time keeping in mind the three categories of family functioning (highly functional, moderately functional and severely dysfunctional), with regard to our seventh research hypothesis, the results showed that students who can count on a highly functional family are those who refer to greater belonging and tangible support, followed by those with moderate function, while students from homes with severe family dysfunction had less appraisal support. Previous scientific literature supports the same relationship between family function and social support [49], and between social support and adolescent adjustment [51,52,53,54]. Thus, as demonstrated by Sánchez-Nuñez et al. [74], parents exert an extremely strong influence on emotional development, forging the image of their children with respect to their own emotional skills by emotional socialization, and are essential in the psychosocial adjustment of adolescents in different environments [75].

We should not leave out mention of some limitations of this study. In the first place, the instruments employed to collect information were self-report measures and could incur social desirability biases, and although it is a widely accepted evaluation technique, it could be completed with other psychometric strategies, such as observation, and involving significant others in the adolescent’s setting, such as parents. In addition, following Piqueras [76], the EQ-i questionnaire uses the same items for the evaluation of the entire adolescent period and childhood, without taking into account the characteristics of each stage according to developmental psychology. One other limitation of this study is its cross-sectional nature, which does not allow causal relationships between variables to be established. Therefore, future studies that analyze the variable present should be performed using qualitative methods, such as observation or interviews of those involved (both the adolescents themselves and their parents and peers). Similarly, to overcome the limitations of cross-sectional studies, longitudinal studies would be suitable for enquiring into the effects of social support, family functionality and emotional intelligence on their wellbeing and psychological adjustment in later stages.

## 5. Conclusions

There are different stages in adolescent development. Papilia, Duskin, and Feldman [77] and UNICEF [2] state the existence of early adolescence (up to 13 years old) and late adolescence (from 14 to 17 or 19 years old), while other authors suggest three different periods: early, middle and late adolescence [78]. However, regardless of the position we start from, it is clear that adolescence spans different stages, and therefore, in the future, the role of age could continue to be explored, both in the development of emotional intelligence and in social support, and its relationship with family function, considering the evolutionary trajectory of girls and boys at the same time. Thus, it could be confirmed whether the small differences contributed by gender to the development of emotional intelligence and perceived social support would affect family function throughout adolescence and whether these differences are maintained over time. Moreover, another future line of research might be a detailed analysis of the relationships between the variables with attention to relevant theoretical models.

In addition, it would be advisable for knowledge of oneself in the emotional terrain (how we feel, what feelings differentiate us from others and how others feel) to be fortified, and a positive mood cultivated and developed. Both can be associated with a functional family environment based on communication, dialogue, affective participation and making use of family resources, as well as the perception of a satisfactory, positive adolescent social support network. These, which would guide both thought and action more appropriately, would probably be better adjusted to the objectives pursued in a particular context.

One of the most important findings of this study is the strong relationship between family function and the emotional intelligence mood dimension. These results emphasize the importance of the family in emotional development and adolescence. Family function and its relationship with social support should also be considered, especially with appraisal support, referring to advice and orientation by the family on subjects of interest or that worry the adolescent, and its lack could be associated with dysfunctional homes. These data advise reinforcement of the emotional health of the family during this stage, by means of awareness programs for family members on adolescent needs that could be carried out from the socio-educational environment (workshops for parents or courses for families of adolescents who attend a high school, for example). In addition to reinforcing the development of emotional intelligence in early adolescence, especially concerning the intrapersonal dimensions and mood, it can also contribute to the configuration of an adjusted social network and a positive, satisfactory perception of social support during adolescence.

## Figures and Tables

**Table 1:** Emotional intelligence by gender. Descriptive statistics and Student’s *t*-test.

EI Factor	Total			Gender						*t*	*p*	95% CI	*d*
				Boys			Girls						
	*n*	*Mean*	*SD*	*n*	*Mean*	*SD*	*n*	*Mean*	*SD*				
Intrapersonal	1287	8.95	3.05	606	9.12	3.03	681	8.80	3.07	1.88	0.060	−0.013, 0.656	0.11
Interpersonal	1287	11.61	2.18	606	11.18	2.17	681	11.99	2.11	−6.80 ***	0.000	−1.049, −0.579	0.38
Stress Management	1287	11.20	2.88	606	11.48	2.82	681	10.95	2.91	3.30 **	0.001	0.216, 0.846	0.18
Adaptability	1287	11.27	2.44	606	11.47	2.37	681	11.09	2.49	2.81 **	0.005	0.117, 0.651	0.16
Mood	1287	11.37	3.23	606	11.94	3.00	681	10.87	3.34	6.08 ***	0.000	0.727, 1.427	0.34

** *p* < 0.01; *** *p* < 0.001.

**Table 2:** Interpersonal support by gender. Descriptive statistics and Student’s *t*-test.

Interpersonal Support	Total			Gender						*t*	*p*	95% CI	*d*
				Boys			Girls						
	*n*	*Mean*	*SD*	*n*	*Mean*	*SD*	*n*	*Mean*	*SD*				
Appraisal Support	1287	12.60	2.74	606	12.25	2.65	681	12.91	2.79	−4.33 ***	0.000	−0.959, −0.361	0.24
Belonging Support	1287	12.96	2.63	606	12.62	2.71	681	13.27	2.52	−4.39 ***	0.000	−0.933, −0.357	0.25
Tangible Support	1287	12.72	2.46	606	12.40	2.39	681	13.01	2.48	−4.44 ***	0.000	−0.875, −0.339	0.25

*** *p* < 0.001.

**Table 3:** Emotional intelligence factors, interpersonal support dimensions and family function. Pearson’s correlations.

Variables	Interpersonal	Stress Management	Adaptability	Mood	Appraisal Support	Belonging Support	Tangible Support	Family Function
Intrapersonal	0.207 ***	−0.034	0.253 ***	0.376 ***	0.272 ***	0.193 ***	0.137 ***	0.292 ***
Interpersonal		−0.013	0.408 ***	0.140 ***	0.158 ***	0.142 ***	0.183 ***	0.136 ***
Stress Management			−0.046	0.128 ***	0.092 **	0.077 **	0.096 **	0.148 ***
Adaptability				0.343 ***	0.146 ***	0.174 ***	0.200 ***	0.166 ***
Mood					0.290 ***	0.294 ***	0.233 ***	0.414 ***
Appraisal Support						0.525 ***	0.476 ***	0.274 ***
Belonging Support							0.557 ***	0.218 ***
Tangible Support								0.227 ***

** *p* < 0.01; *** *p* < 0.001.

**Table 4:** Means and standard deviations corresponding to the Family Function Groups (IV) in the interpersonal support and emotional intelligence dimensions.

Variables	Family Function											
	Severely Dysfunctional			Moderately Functional			Highly Functional					
	*n*	*M*	*SD*	*n*	*M*	*SD*	*n*	*M*	*SD*	F	*p*	*η_p_²*
Appraisal Support	114	10.87	3.32	281	12.16	2.72	891	12.97	2.56	36.07	0.000	0.053
Belonging Support	114	11.59	3.11	281	12.51	2.70	891	13.29	2.46	27.51	0.000	0.041
Tangible Support	114	11.38	2.83	281	12.30	2.56	891	13.03	2.30	29.19	0.000	0.044
Intrapersonal	114	7.15	2.61	281	8.29	3.03	891	9.39	2.99	37.56	0.000	0.055
Interpersonal	114	11.24	2.49	281	11.27	2.15	891	11.76	2.13	7.28	0.001	0.011
Stress Management	114	10.18	3.07	281	10.62	2.90	891	11.51	2.80	18.23	0.000	0.028
Adaptability	114	10.67	2.72	281	10.89	2.47	891	11.47	2.37	9.79	0.000	0.015
Mood	114	8.21	3.33	281	10.36	3.03	891	12.09	2.94	104.73	0.000	0.140

## Data Availability

The data that support the findings of this study are available from the corresponding author upon reasonable request.

## References

[1] Meeus W. (2016). Adolescent psychosocial development: A review of longitudinal models and research. Dev. Psychol..

[2] UNICEF (2011). Adolescence: An Age of Opportunity.

[3] Cánovas P., Sahuqillo P. (2014). Familias y Menores. Retos y Propuestas Pedagógicas [Families and Minors. Challenges and Pedagogical Proposals].

[4] Musitu G., Román J.M., Gutiérrez M. (1996). Educación Familiar y Socialización de Los Hijos [Family Education and Socialization of Children].

[5] Nardone G., Gianonotti E., Rocchi R. (2003). Modelos de Familia. Conocer y Resolver Los Problemas Entre Padres e Hijos [Family Models. Know and Solve Problems between Parents and Children].

[6] Nye F.I., Bahr S., Carlson J.E., Gecas V., Maclaughlin S., Slocum W.L. (1976). Role Structure And Analysis Of The Family.

[7] Hamarta E., Deniz M., Saltali N. (2009). Attachment Styles as a Predictor of Emotional Intelligence. Educ. Sci. Theory Pract..

[8] De la Fuente I.I., Rodríguez-Fernández A., Escalante N. (2019). Medida del apoyo social percibido (APIK) en la adolescencia [Measurement of perceived social support (APIK) in adolescence]. Eur. J. Investig. Health Psychol. Educ..

[9] Fernández O., Ramos E., Goñi E., Rodríguez A. (2020). The role of social support in school adjustment during secondary education. Psicothema.

[10] Ozbay F., Johnson D.C., Dimoulas E., Morgan C.A., Charney D., Southwick S. (2007). Social support and resilience to stress: From neurobiology to clinical practice. Psychiatry.

[11] Cohen S., Hoberman H.M. (1983). Positive events and social supports as buffers of life change stress 1. J. Appl. Soc. Psychol..

[12] Cohen S., Mermelstein R., Kamarck T., Hoberman H.M., Sarason I.G., Sarason B.R. (1985). Measuring the functional components of social support. Social Support: Theory, Research and Applications.

[13] Ciarrochi J., Heaven P.C. (2008). Learned social hopelessness: The role of explanatory style in predicting social support during adolescence. J. Child. Psychol. Psychiatr..

[14] Rowsell H.C., Ciarrochi J., Heaven P.C., Deane F.P. (2014). The role of emotion identification skill in the formation of male and female friendships: A longitudinal study. J. Adolesc..

[15] Von Salisch M., Lüpschen N., Kanevski R. (2013). Having and losing friends: Necessary social-emotional competencies in adolescents. Prax Kinderpsychol. Kinderpsychiatr..

[16] Orcasita L.T., Uribe A.F. (2010). La importancia del apoyo social en el bienestar del adolescente [The importance of social support in the well-being of the adolescent]. Psychol. Av. Discip..

[17] Hogan M.J., Parker J.D., Wiener J., Watters C., Wood L.M., Oke A. (2010). Academic success in adolescence: Relationships among verbal IQ, social support and emotional intelligence. Aust. J. Psychol..

[18] Bar-On R. (1997). The Emotional Quotient Inventory (EQ-i): Technical Manual.

[19] Salovey P., Mayer J.D. (1990). Emotional intelligence. Imagin. Cogn. Pers..

[20] Gardner H. (1992). Multiple Intelligences.

[21] Goleman D. (1995). Emotional Intelligence.

[22] Bar-On R. (1996). EQ-i: EQ-Inventory: Item Booklet.

[23] Petrides K.V., Furnham A. (2001). Trait emotional intelligence: Psychometric investigation with reference to established trait taxonomies. Eur. J. Personal..

[24] Petrides K.V., Mikolajczak M., Mavroveli S., Sánchez-Ruiz M.J., Furnham A., Pérez-González J.C. (2016). Developments in Trait Emotional Intelligence Research. Emot. Rev..

[25] Pérez-González J.C., Saklofske D.H., Mavroveli S. (2020). Editorial: Trait Emotional Intelligence: Foundations, Assessment, and Education. Front. Psychol..

[26] O’Connor P.J., Hill A., Kaya M., Martin B. (2019). The Measurement of Emotional Intelligence: A Critical Review of the Literature and Recommendations for Researchers and Practitioners. Front. Psychol..

[27] Esnaola I., Arias V.B., Freeman J., Wang Y., Arias B. (2018). Validity evidence based on internal structure of scores of the emotional quotient inventory: Youth version short (EQ-i: YV-S) in a Chinese sample. J. Psychoeduc. Assess..

[28] Puertas P., Zurita-Ortega F., Chacón-Cuberos R., Castro-Sánchez M., Ramírez-Granizo I., González G. (2020). Emotional intelligence in the educational field: A meta-analysis. An. Psicol. Spain.

[29] Esnaola I., Azpiazu L., Antonio-Agirre I., Sarasa M., Ballina E. (2018). Validity evidence of Emotional Quotient Inventory: Youth Version (Short) in a sample of Mexican adolescents. Estud. Psicol..

[30] Pérez-Fuentes M.C., Molero M.M., Oropesa N.F., Simón M.M., Gázquez J.J. (2019). Relationship between digital creativity, parenting style and adolescent performance. Front. Psychol..

[31] Baumgartner F. (2009). Social intelligence in relation with interpersonal traits. Cesk Psychol..

[32] Silveri M.M., Tzilos G.K., Pimentel P.J., Yurgelun-Todd D.A. (2004). Trajectories of adolescent emotional and cognitive development: Effects of gender and risk for drug use. Ann. N. Y. Acad. Sci..

[33] Uribe A.F., Orcasita L., Gómez E.A. (2012). Bullying, redes de apoyo social y funcionamiento familiar en adolescentes de una institución educativa de Santander, Colombia [Bullying, social support networks and family functioning in adolescents from an educational institution in Santander, Colombia]. Psychol. Av. Discip..

[34] Barraca J., López-Yarto L. (1996). ESFA. Escala de Satisfacción Familiar Por Adjetivos [ESFA. Family Satisfaction Scale by Adjectives].

[35] Cracco C., Costa-Ball C.D. (2019). Propiedades Psicométricas de la Escala de Comunicación Familiar. Rev. Iberoam Diagn. Eval. Psicol..

[36] Epstein N.B., Baldwin L.M., Bishop D.S. (1983). The McMaster Family Assessment Device. J. Marital. Fam. Ther..

[37] Quintanilla G.T., Sotomayor M.D.P.D.L., Hernández O.M., Peralta P.C., Domingo M.M., Roque A.H., Coqui M.L. (2013). Escala de Satisfacción Familiar por Adjetivos (ESFA) en escolares y adolescentes mexicanos: Datos normativos [Family Satisfaction Scale by Adjectives (ESFA) in Mexican schoolchildren and adolescents: Normative data]. Salud. Ment..

[38] Martínez-Pampliega A., Merino L., Iriarte L., Olson D.H. (2017). Psychometric properties of the Spanish version of the Family Adaptability and Cohesion Evaluation Scale IV. Psicothema.

[39] Olson D.H. (2011). FACES IV and the circumplex model: Validation study. J. Marital. Fam. Ther..

[40] Olson D., Barnes H. (2010). Family Communication Scale.

[41] Olson D., Gorall D., Tiesel J. (2006). FACES IV Package. Administration Manual.

[42] Tsamparli A., Petmeza I., McCarthy G., Adamis D. (2018). The Greek version of the mcmaster family assessment device. Psych. J..

[43] Villarreal-Zegarra D., Copez-Lonzoy A., Paz-Jesús A., Costa-Ball C.D. (2017). Validez y confiabilidad de la Escala Satisfacción Familiar en estudiantes universitarios de Lima Metropolitana, Perú [Validity and reliability of the Family Satisfaction Scale in university students of Metropolitan Lima, Peru]. Actual. Psicol..

[44] Smilkstein G. (1978). The family APGAR: A proposal for a family function test and its uses by physicians. J. Fam. Pract..

[45] Bellón J.A., Delgado A., Luna J.D., Lardelli P. (1996). Validez y fiabilidad del cuestionario de función familiar Apgar-Familiar [Validity and reliability of the Apgar-Familial family function questionnaire]. Aten. Primaria.

[46] Smilkstein G., Ashworth C., Montano D. (1982). Validity and reliability of the Family APGAR as a test of family function. J. Fam. Pract..

[47] Freed R.D., Rubenstein L.M., Daryanani I., Olino T.M., Alloy L.B. (2016). The relationship between family function and adolescent depressive symptoms: The role of emotional clarity. J. Youth Adolesc..

[48] Pérez-Fuentes M.C., Molero M.M., Barragán A.B., Gázquez J.J. (2019). Family function, emotional intelligence, and values: Analysis of the relationship with aggressive behavior in adolescents. Int. J. Environ. Res. Public Health.

[49] Pérez A.M., Pérez R.M., Martínez M.F., Leal F.H., Mesa I.G., Jiménez I.P. (2007). Family structure and function during adolescence: Relationship with social support, tobacco, alcohol and drugs consumption, and psychic discomfort. Aten. Primaria.

[50] Torrel M., Delgado M. (2016). Funcionamiento familiar y depresión en adolescentes de la IE Zarumilla-Tumbes, 2013 [Family functioning and depression in adolescents from EI Zarumilla-Tumbes, 2013]. Cienc. Desarro..

[51] Cutrín O., Maneiro L., Sobral J., Gómez-Fraguela J.A. (2019). Longitudinal effects of parenting mediated by deviant peers on violent and non-violent antisocial behaviour and substance use in adolescence. Eur. J. Psychol. Appl. Leg Context..

[52] Gázquez J.J., Pérez-Fuentes M.C., Molero M.M., Barragán A.B., Martos Á., Sánchez-Marchán C. (2016). Drug use in adolescents in relation to social support and reactive and proactive aggressive behavior. Psicothema.

[53] Olivares-Olivares P.J., Ortiz-González P.F., Olivares J. (2019). Role of social skills training in adolescents with social anxiety disorder. Int. J. Clin. Health Psychol..

[54] Rodríguez A., Antonio I., Ramos E., Revuelta L. (2020). The role of affect-communication and rule setting in perceived family support and school adjustment. Eur. J. Educ. Psychol..

[55] Muñiz-Rivas M., Vera M., Povedano-Díaz A. (2019). Parental Style, Dating Violence and Gender. Int. J. Environ. Res. Public Health.

[56] Šumskas L., Zaborskis A. (2017). Family Social Environment and Parenting Predictors of Alcohol Use among Adolescents in Lithuania. Int. J. Environ. Res. Public Health.

[57] Machimbarrena J.M., González-Cabrera J., Garaigordobil M. (2019). Family Variables Related to Bullying and Cyberbullying: A Systematic Review. Pensam Psicol..

[58] Martínez-Ferrer B., León-Moreno C., Musitu-Ferrer D., Romero-Abrio A., Callejas-Jerónimo J.E., Musitu-Ochoa G. (2019). Parental Socialization, School Adjustment and Cyber-Aggression among Adolescents. Int. J. Environ. Res. Public Health.

[59] Ul Ain N., Munir M., Suneel I. (2021). Role of emotional intelligence and grit in life satisfaction. Heliyon.

[60] Ciarrochi J., Heaven P.C., Supavadeeprasit S. (2008). The link between emotion identification skills and socio-emotional functioning in early adolescence: A 1-year longitudinal study. J. Adolesc..

[61] Azpiazu L., Esnaola I., Sarasa M. (2015). Capacidad predictiva del apoyo social en la inteligencia emocional de adolescentes [Predictive capacity of social support in the emotional intelligence of adolescents]. Eur. J. Educ. Psychol..

[62] Alghamdi N.G., Aslam M., Khan K. (2017). Anger and interpersonal relationships: Social life in adolescence. Eur. Online J. Nat. Soc. Sci..

[63] Bar-On R., Parker J.D.A. (2000). Emotional Quotient Inventory: Youth Version (EQ-i:YV): Technical Manual.

[64] Pérez-Fuentes M.C., Gázquez J.J., Mercader I., Molero M.M. (2014). Brief Emotional Intelligence Inventory for Senior Citizens (EQ-i-M20). Psicothema.

[65] IBM Corp. (2017). IBM SPSS Statistics for Windows, Version 25.0.

[66] Skovlund E., Fenstad G.U. (2001). Should we always choose a nonparametric test when comparing two apparently nonnormal distributions?. J. Clin. Epidemiol..

[67] Fagerland M.W., Sandvik L. (2009). Performance of five two-sample location tests for skewed distributions with unequal variances. Contemp Clin. Trials.

[68] Cohen J. (1988). Statistical Power Analysis for the Behavioral Sciences.

[69] McDonald R.P. (1999). Test. Theory: Un Enfoque Unificado.

[70] Ventura-León J.L., Caycho T. (2017). El coeficiente Omega: Un método alternativo para la estimación de la confiabilidad [The Omega Coefficient: An Alternative Method for Estimating Reliability]. Rev. Lat. Cienc Soc. Niñez Juv..

[71] Toscano-Hermoso M.D., Ruiz-Frutos C., Fagundo-Rivera J., Gómez-Salgado J., García-Iglesias J.J., Romero-Martín M. (2020). Emotional Intelligence and Its Relationship with Emotional Well-Being and Academic Performance: The Vision of High School Students. Children.

[72] Garaigordobil M. (2020). Intrapersonal Emotional Intelligence during Adolescence: Sex Differences, Connection with other Variables, and Predictors. Eur. J. Investig. Health Psychol. Educ..

[73] Núñez-Fadda S.M., Castro-Castañeda R., Vargas-Jiménez E., Musitu-Ochoa G., Callejas-Jerónimo J.E. (2020). Bullying Victimization among Mexican Adolescents: Psychosocial Differences from an Ecological Approach. Int. J. Environ. Res. Public Health.

[74] Sánchez-Núñez M., García-Rubio N., Fernández-Berrocal P., Latorre J.M. (2020). Emotional Intelligence and Mental Health in the Family: The Influence of Emotional Intelligence Perceived by Parents and Children. Int. J. Environ. Res. Public Health.

[75] Povedano-Diaz A., Muñiz-Rivas M., Vera-Perea M. (2020). Adolescents’ Life Satisfaction: The Role of Classroom, Family, Self-Concept and Gender. Int. J. Environ. Res. Public Health.

[76] Piqueras J.A., Salvador M.d.C., Soto-Sanz V., Mira F., Pérez-González J.-C. (2020). Strengths Against Psychopathology in Adolescents: Ratifying the Robust Buffer Role of Trait Emotional Intelligence. Int. J. Environ. Res. Public Health.

[77] Papalia D.E., Duskin R., Feldman R.D. (2013). Desenvolvimento Humano.

[78] Wong D.L., Hockenberry M.J., Wilson D. (2011). Wong’s Nursing Care of Infants and Children.

